# Amino acid deprivation in cancer cells with compensatory autophagy induction increases sensitivity to autophagy inhibitors

**DOI:** 10.1080/23723556.2024.2377404

**Published:** 2024-07-14

**Authors:** Takahito Fukui, Manami Yabumoto, Misuzu Nishida, Shiori Hirokawa, Riho Sato, Taichi Kurisu, Miyu Nakai, Md. Abul Hassan, Koji Kishimoto

**Affiliations:** a Division of Bioscience and Bioindustry, Tokushima University Graduate School of Sciences and Technology for Innovation, Tokushima, Japan; b Graduate School of Environment and Energy Engineering, Waseda University, Tokyo, Japan; c Graduate School of Medical Life Science, Yokohama City University, Yokohama, Japan; d Faculty of Bioscience and Bioindustry, Tokushima University Graduate School of Advanced Technology and Science, Tokushima, Japan; e Division of Bioscience and Bioindustry, Tokushima University Graduate School of Technology, Industrial and Social Sciences, Tokushima, Japan

**Keywords:** Amino acid deprivation, amino acid transporter, autophagy, cancer, chemoresistance

## Abstract

Inhibition of autophagy is an important strategy in cancer therapy. However, prolonged inhibition of certain autophagies in established cancer cells may increase therapeutic resistance, though the underlying mechanisms of its induction and enhancement remain unclear. This study sought to elucidate the mechanisms of therapeutic resistance through repeated autophagy inhibition and amino acid deprivation (AD) in an in vitro model of in vivo chronic nutrient deprivation associated with cancer cell treatment. In the human cervical cancer cell line HeLa and human breast cancer cell line MCF-7, initial extracellular AD induced the immediate expression of endosomal microautophagy (eMI). However, repeated inhibition of eMI with U18666A and extracellular AD induced macroautophagy (MA) to compensate for reduced eMI, simultaneously decreasing cytotoxicity. Here, hyperphosphorylated JNK was transformed into a hypophosphorylated state, suggesting conversion of the cell death signal to a survival signal. In a nutrient medium, cell death could not be induced by MA inhibition. However, since LAT1 inhibitors induce intracellular AD, combining them with MA and eMI inhibitors successfully promoted cell death in resistant cells. Our study identified a novel therapeuic approach for promoting cell death and addressing therapeutic resistance in cancers under autophagy-inhibitor treatment.

## Graphical abstract

**Figure:**
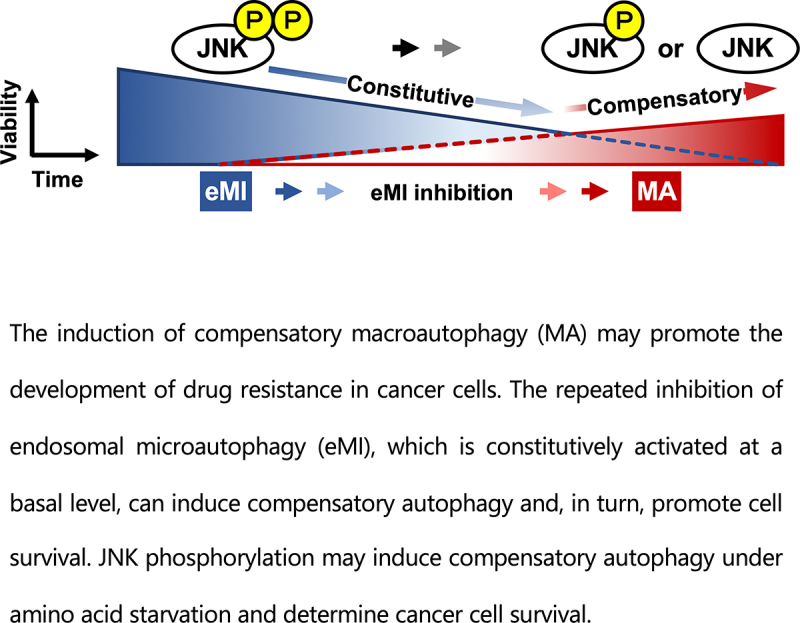

## Introduction

Autophagy is an intracellular process that maintains energy and cellular homeostasis during metabolic stress or nutrient deprivation by inducing lysosomal degradation and recycling excess or damaged cellular organelles, proteins, and unwanted cytosolic components.^1^ Lysosomes participate in three forms of autophagy, including macroautophagy (MA, a nonselective autophagy), endosomal microautophagy (eMI), and chaperone-mediated autophagy.^2^ Mammalian target of rapamycin complex 1 (mTORC1), a key regulator of MA,^3^ is inactivated by intracellular amino acid deprivation (AD) and participates in the nonselective MA-related sensing of intracellular AD, induced in a delayed manner.^2,4^

Autophagosomes (bilayer structures containing MA substrates) fuse with lysosomes to form autolysosomes, but may also fuse with late endosomes to form amphisomes and ultimately autolysosomes.^5^ Most proteins involved in these fusion events, such as SNARE and RAB7, also participate in the endocytosis pathway.^5^ Thus, MA and eMI may act synergistically in this process.^6^ However, it is unclear how these forms of autophagy contribute to autophagy inhibitor resistance in cancer cells, where autophagy plays a dual role: it inhibits cancer development but also serves as a survival mechanism in the nutrient-poor environments^7^ of rapidly proliferating and metabolizing cancer cells. In this environment, amino acids are essential for cell survival and proliferation because they not only signal the nutritional status of cells but are also intimately involved in nucleic acid and protein synthesis to maintain biomass, energy supply, and cellular redox homeostasis.^8^ Excessive proliferation of cancer cells leads to abnormal and immature vascular structures within the tumor, causing the tumor to fall into chronic blood flow insufficiency. As a result, cancer cells often experience localized AD.^9^ Therefore, cancer cells are dependent on autophagy as a growth-promoting mechanism, unlike in normal cells where autophagy serves as a defense mechanism for maintaining homeostasis.^10^

Autophagy inhibition has attracted widespread attention for selectively inducing cell death in cancer cells.^11^ Chloroquine (CQ) and hydroxychloroquine (HCQ), which inhibit the final step of autophagy by preventing lysosomal acidification, have shown promising results in a clinical trial.^11^ However, these inhibitors exhibit dose-dependent toxicity both in vitro and in vivo, limiting their use as single agents. To address this limitation, combination therapies using autophagy inhibitors and conventional anticancer drugs have been explored. Unfortunately, a phase I trial of combination therapy using HCQ and the cytotoxic anticancer drug temozolomide reported progressive brain metastasis after 4 months of treatment,^12^ and a phase I trial of HCQ in combination with the molecularly targeted drug bortezomib showed cancer progression in 45% of patients after 9–14 weeks.^13^ These combination therapies may also induce inflammatory cytokine activity, such as macrophage migration inhibitory factor and interleukin-6 expression in triple-negative breast cancer cells,^14^ increasing the potential for chronic inflammation. When used long-term in combination with certain classes of anticancer drugs, autophagy inhibitors may interfere with the immediate and long-term efficacy of the drug, highlighting serious issues such as side effects and drug resistance.^14,15^ A major concern is that long-term treatment conversely and inevitably selects for resistant cell lineages that are characteristic of refractory cancers,^16^ such as subpopulations of dormant cancer cells^17^ and stem cells induced by reprogramming of this subpopulation.^18^ Cell survival in this resistant lineage is primarily impeded by intracellular oxidation and impaired energy delivery. However autophagy enhances antioxidant capacity and high energy efficiency through the mitophagic removal of damaged mitochondria and mitochondrial biogenesis, which complicates cancer treatment and increases the risk of disease progression and metastasis.^19^ The inhibition of autophagy has been effective in eliminating some dormant cells and resistant lineages and preventing recurrence.^20^ However, the limited efficacy of single inhibitors or combination therapies with anticancer agents and the severe adverse outcomes reported in most studies emphasize the need for further research and development.

This study investigated the survival mechanisms adopted by cancer cells exposed to repeated treatment with U18666A^21^ and AD, which selectively accumulate cholesterol in the membranes of late endosomes and lysosomes, and evaluated potential approaches for inhibiting this mechanism.

## Results

### Acute phase autophagy via extracellular AD in HeLa and MCF-7 cells operates via an autophagy pathway distinct from MA

**Figure 1.**
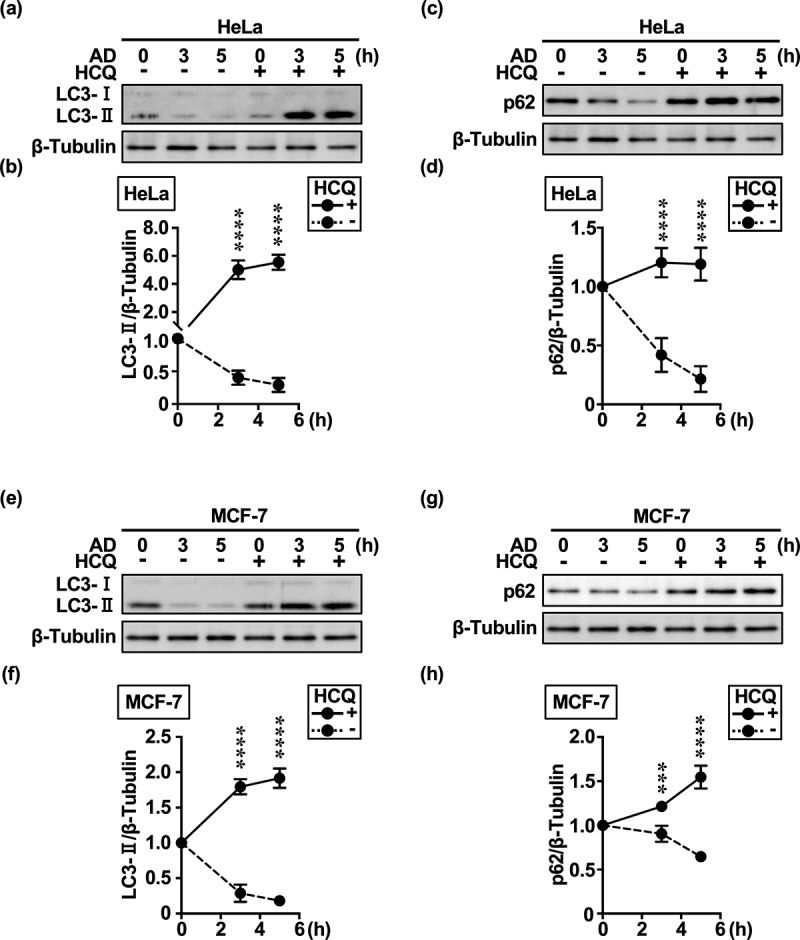
HeLa and MCF-7 cells rapidly degrade LC3-II and p62 under extracellular AD. (a, c, e, g) Western blot images of LC3-II and p62 expression over time in response to extracellular AD in HeLa and MCF-7 cells. Western blot images were generated using 3 μg of cell extracts per well to evaluate changes in the amount of degradation and accumulation of LC3-II (a, e) and p62 (c, g) after AD in the absence or presence of HCQ (100 μM). (b, d, f, h) Quantification of LC3-II (b, f) and p62 (b, h) degradation (dashed lines) and accumulation (solid lines) over time. Results are expressed as the mean ± SD of three independent experiments. ***p < .001, ****p < .0001 (two-way ANOVA with Sidak’s multiple comparisons test). AD, amino acid deprivation; HCQ, hydroxychloroquine.

In HeLa and MCF-7 cells, the expressions of the autophagosome marker LC3-II (Figure 1a,b,e,f; Supplementary Figure S1a,b,e,f) and selective MA receptor p62/SQSTM1 (p62) (Figure 1c,d,g,h; Supplementary Figure S1c,d,g,h) were reduced to ≤ 50% at the time of or 5 h after extracellular AD. These autophagy substrates accumulated upon treatment with the late autophagy inhibitor HCQ (Figure 1) and lysosomal V-ATPase inhibitor concanamycin A (ConA) [Supplementary Figure S1] up to 5 h after treatment. Although we cannot exclude the immediate degradation of p62 by the nonselective MA pathway, this would be unexpected as it is more commonly degraded in a delayed manner via intracellular AD-induced mTORC1 inhibition.^22^

As LC3-II is not involved in chaperone-mediated autophagy^23^ and its lipidation establishes endosome-selective membrane turnover in addition to autophagosome membrane formation and maturation,^24^ our results suggest the involvement of an endosomal-mediated autophagy pathway, at least in HeLa and MCF-7 cells. Therefore, to investigate whether substrate degradation via immediately induced autophagy is mediated by the endosomal network reaction pathway, we treated cells with U18666A,^21^ an inhibitor of eMI and potent inhibitor of intracellular cholesterol transport.

### Endosomal microautophagy may participate in acute phase autophagy via extracellular AD in HeLa and MCF-7 cells

**Figure 2.**
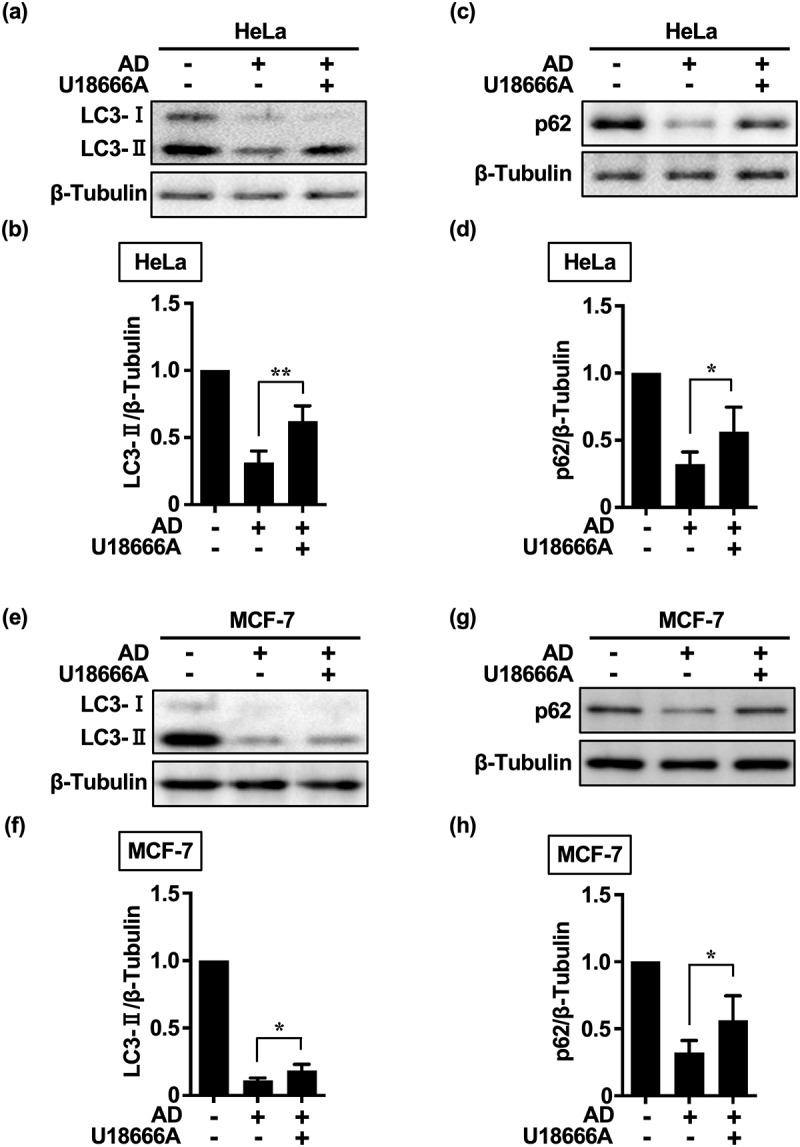
HeLa and MCF-7 cells induce endosome-mediated microautophagy under AD.

Cholesterol transport has been implicated in eMI.^25^ U18666A accumulates cholesterol in organelle membranes of the endolysosomal system and inhibits essential cellular functions through membrane stabilization and internalization of cell surface proteins and lipids, thereby inhibiting multivesicular body (MVB) dynamics and ultimately eMI.^21^ To clarify the effect of U18666A on cholesterol homeostasis and ensure that only intracellular cholesterol affected the in vitro outcomes, no serum was administered from the time of drug administration and U18666A was administered before AD. When HeLa and MCF-7 cells were treated with U18666A, an immediate and significant accumulation of LC3-II and p62 was observed at 5 h (Figure 2). These results suggest that eMI may be responsible for immediate autophagy in HeLa and MCF-7 cells.

(a, e, c, g) Western blot images of LC3-II (a, e) and p62 expression (c, g) in cells exposed to U18666A (HeLa cells, 5 μg/mL; MCF-7 cells, 8 μg/mL) for 3 h followed by washing and culturing with EBSS for 5 h. (b, f, d, h) Quantification of LC3-II (b, f) and p62 (c, d) degradation and accumulation. Results are expressed as the mean ± SD of three independent experiments. *p < .05, **p < .01 (one-way ANOVA with Tukey’s multiple comparisons test). AD, amino acid deprivation.

However, the accumulation of LC3-II following U18666A treatment was greater in HeLa cells than in MCF-7 cells (Figure 2a,b,e,f). This suggests that Beclin-1 haploinsufficiency in MCF-7 cells^26^ may affect eMI activity, given that Beclin-1 promotes endocytotic trafficking by forming a complex with UV radiation resistance associate gene (UVRAG),^27^ thereby inducing immediate autophagy. As inhibition of cholesterol transport may affect MA flux by preventing autophagosome – lysosome fusion,^28^ the accumulation of p62 and LC3-II by U18666A may also be due to the inhibition or loss of MA flux. Moreover, the intracellular levels of autophagy marker proteins were higher at the onset of AD [immediately after changing Dulbecco’s modified Eagle’s medium (DMEM) containing 10% serum to Earle’s balanced salt solution (EBSS)] and under non-AD conditions (DMEM containing 10% serum).

### Inhibition of eMI, which is acutely activated by extracellular AD, induces compensatory autophagy of pathways distinct from eMI

To determine the extent to which MA contributes to immediate autophagy via extracellular AD, we indirectly quantified the contribution of compensatory MA by silencing the VPS4B isoform^29^ of VPS4,^25,30,31^ a regulatory ATPase of endosomal sorting complexes required for transport (ESCRT)-III. As silencing of both VPS4A and VPS4B can effectively block eMI,^25^ we selected the VPS4B sequence, which may partially silence VPS4A (Supplementary Figure S2a). As the siRNA siVPS4 was able to deplete VPS4A/B proteins to nearly undetectable levels at 50 nM (Supplementary Figure S2b), we performed validation tests using 50 nM siVPS4. Treatment with siVPS4 resulted in LC3-II accumulation, as the flux of eMI and MA under AD was completely stopped by the lysosomal inhibitor bafilomycin (BafA1). Approximately 66% of this LC3-II accumulation was derived from eMI inhibited by siVPS4. Consistent with the results in Figure 2, although eMI was preferentially activated in HeLa cells under single AD, the remaining LC3-II content (~34%) was likely associated with autophagy pathways other than eMI.

**Figure 3.**
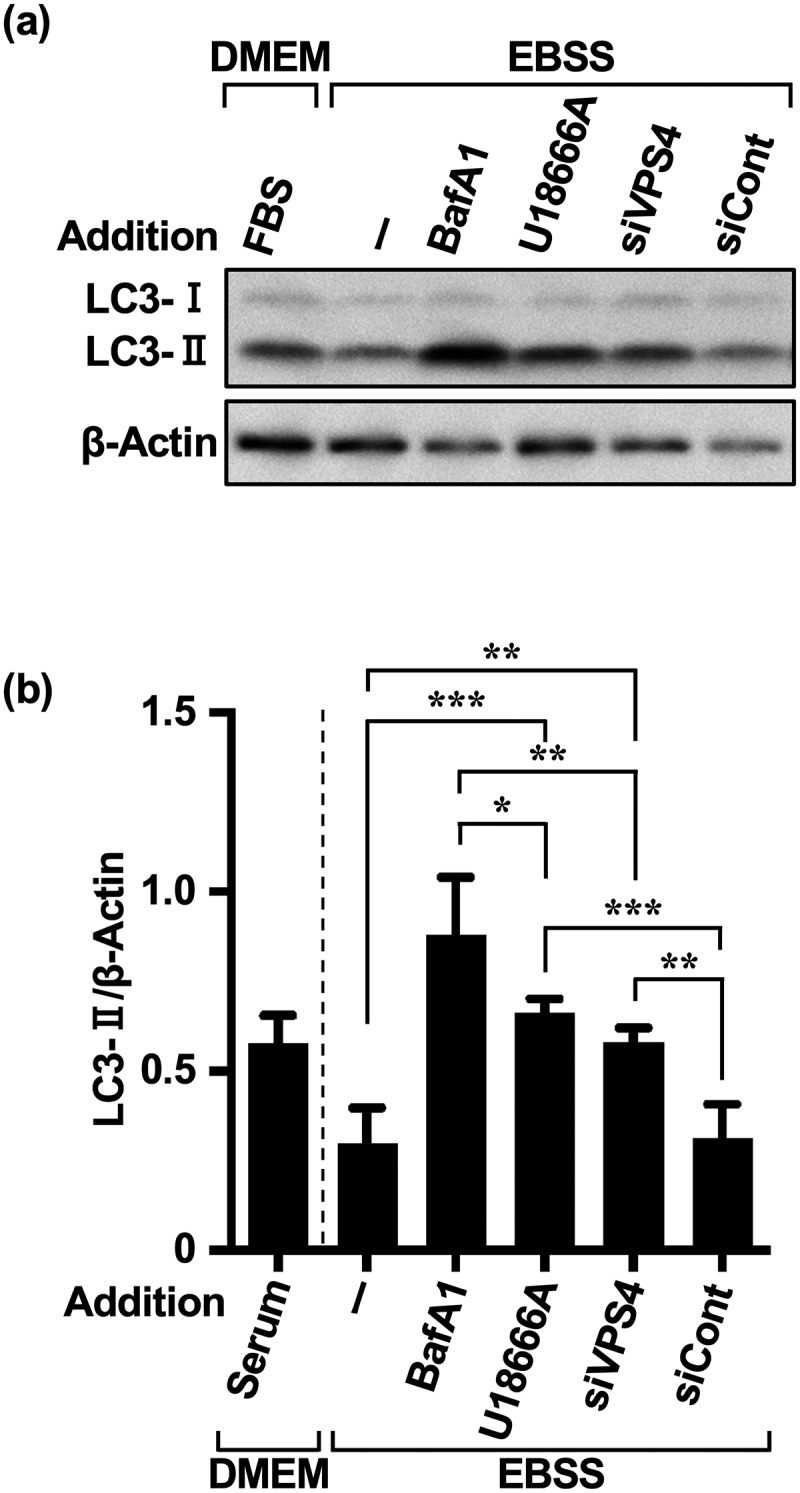
U18666A and AD treatments in HeLa and MCF-7 cells induce a different pathway from eMI. (a) Western blot images of HeLa cells treated with each inhibitor [BafA1 (50 nM), U18666A (5 μg/mL), siVPS4 (50 nM), and siCont (50 nM)] and AD. Cells were pretreated with BafA1 and U18666A for 3 and 1 h, respectively, washed, and exposed to EBSS for 5 h. Non-AD conditions were established using DMEM with 10% serum. (b) Quantification of LC3-II degradation and accumulation. *p < .05, **p < .01, ***p < .001, ****p < .0001 (two-way ANOVA with Tukey’s multiple comparisons test). AD, amino acid deprivation; BafA1, bafilomycin A1; EBSS, Earle’s balanced salt solution.

The accumulation of LC3-II did not differ significantly between the U18666A and siVPS4 treatments (Figure 3). This was consistent with reports showing that the amphisomal pathway comprising autophagosomes and MVB fusion is the major pathway in HeLa cells,^29^ and that U18666A has a more potent inhibitory effect on the endolysosomal system.^25,28,30^ However, although many studies have used U18666A as an eMI inhibitor to block MVB dynamics,^21,25,28,30^ it is only a cholesterol transport inhibitor, and the results have to be interpreted accordingly. We hypothesized that compensatory activated autophagy pathways contribute to the development of cancer cell resistance under repeated treatment with autophagy inhibitors, especially relating to the MA pathway,^5^ which is coordinated with the eMI pathway. As p62 is both a substrate of MA and the most effectively degraded substrate via selective eMI activity during AD,^25,32^ we evaluated the activity of the MA pathway in its crosstalk with the eMI pathway^30^ based on p62 accumulation.

### Repeated treatment with U18666A and AD results in compensatory induction of MA in eMI

We investigated the accumulation of p62^32^ by selective inhibition of class III PI3K (PI3K-C3, VPS34), a major component of the PI3K complex required for autophagosome formation in MA, using wortmannin (Wtn) in HeLa cells under chronic AD after three repeated treatments with U18666A and AD. To verify the specific inhibition of PI3K-C3 as opposed to PI3K-C1 (class I), cells were treated with 10 nM Wtn (IC_50_ 10 nM),^33,34^ representing the low nanomolar range. This can selectively inhibit VPS34, a key component in the crosstalk between the eMI and MA pathways.^30^ Notably, the class II isoform PI3K-C2α is unlikely to be involved in MA, given its low Wtn sensitivity^35^ and inability to generate PtdIns(3,4,5)P3 from PtdIns(4,5)P2.

**Figure 4.**
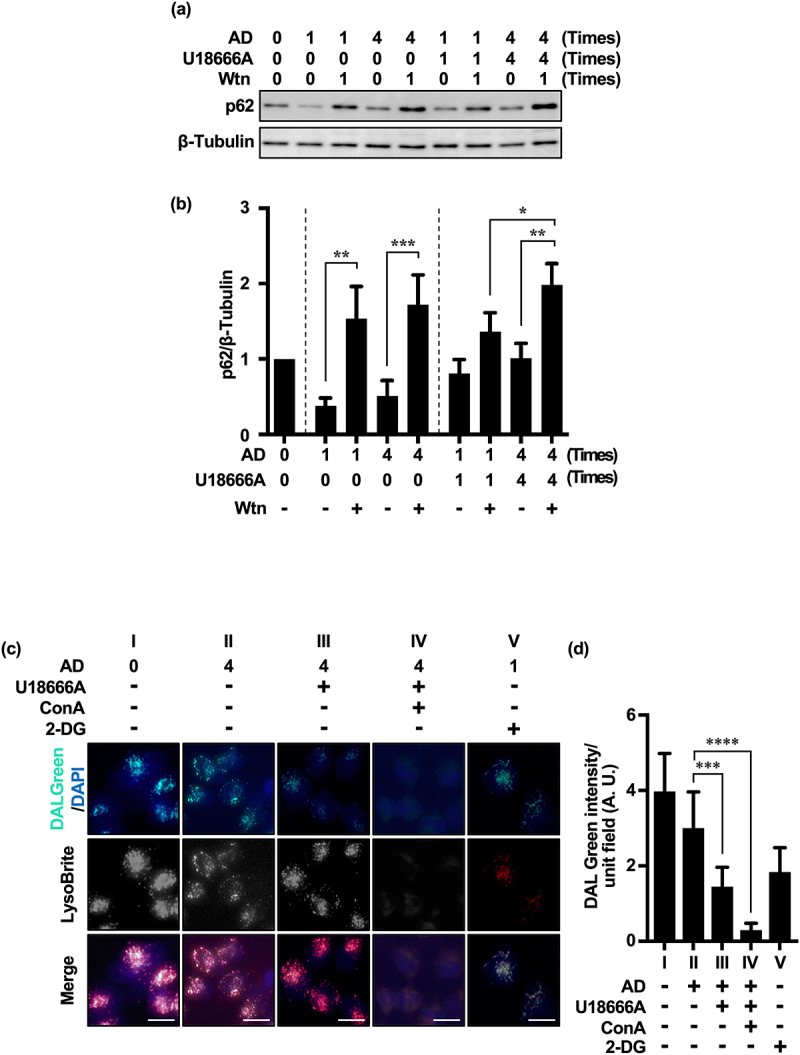
Repeated treatment with U18666A and AD induces MA in HeLa and MCF-7 cells. (a) Western blot image of p62 in HeLa cells after U18666A treatment (first time, 5 μg/mL, 5 h exposure; second and subsequent times, 2 μg/mL, 3 h exposure) followed by washing and 5 h of AD exposure. (b) Quantification of p62 degradation and accumulation. Results are expressed as the mean ± SD of three independent experiments. (c) In the fourth treatment, HeLa cells after three treatments with U18666A and AD were pretreated with EBSS alone (panel II), U18666A (2 μg/mL) and ConA (5 nM) for 3 h, respectively, washed and then treated with EBSS alone (panel III) or EBSS with ConA (panel IV) for 5 h, or pretreated with 2-DG (1 mM) for 3 h followed by EBSS (panel V) for 5 h. Note: panel I shows cells cultured under non-AD conditions (DMEM with 10% serum). Autolysosomes and functional lysosomes are indicated by green and red fluorescence, respectively. (d) Autolysosome-expressing cells as a percentage of total cells. *n* = 126 (panel I), *n* = 132 (panel II), *n* = 120 (panel III), *n* = 126 (panel IV), *n* = 123 (panel V); + and – in the graph footnotes indicate the presence or absence of a fourth U18666A, Wtn, or AD treatment. Results are expressed as the mean ± SD of three independent experiments. *p < .05, **p < .01, ***p < .001, ****p < .0001 (two-way ANOVA with Tukey’s multiple comparisons test). 2-DG, 2-deoxy-D-glucose; AD, amino acid deprivation; ConA, concanamycin A; Wtn, wortmannin.

There was no significant difference in p62 accumulation between single and repeated AD treatments in the presence of Wtn without U18666A treatment. This indicates that in the presence of AD stress alone, even after repeated treatments, the MA flux pathway remained unaffected. However, repeated treatment with AD and U18666A significantly increased p62 accumulation, more so than a single treatment (U18 × 1 + Wtn:U18 × 4 + Wtn = 1.36 ± 0.35:1.98 ± 0.29; Figure 4a,b). These results suggest the activation of the MA pathway by repeated U18666A and AD treatments. Furthermore, considering that there was no difference in p62 accumulation between single and repeated AD treatments in the presence of Wtn, we presumed that U18666A is required for the increase in compensatory MA (Figure 4a,b).

We investigated the effect of U18666A on autolysosome formation using the chemical probe DALGreen (4 μM),^36^ which considers LC3-II kinetics and emits green fluorescence under the acidic conditions of the autolysosome formation phase. With repeated treatment of AD alone, a punctate green fluorescent signal was observed on the red fluorescent signal of LysoBrite, indicating enhanced autolysosome formation (Figure 4c, Panel II, and Figure 4d). However, combined with repeated U18666A treatment, the green fluorescent signal was reduced, suggesting the inhibition of autolysosome formation by U18666A (Figure 4c, panel III, and Figure 4d). U18666A may inhibit the flux of different lysosomal autophagy pathways by inhibiting the fusion between autophagy-related structures, such as amphisomes, and lysosomes; moreover, inhibition of cholesterol transport can prevent lysosomal autophagosome – lysosome fusion and affect MA flux,^25,28^ consistent with our results. Our results suggest that the changes in autophagy substrate accumulation following U18666A treatment may not directly reflect the degree of eMI pathway inhibition. U18666A treatment did not alter the fluorescence intensity of LysoBrite (Figure 4c, Panel III), suggesting that U18666A does not affect lysosomal acidity.^25^ However, the addition of ConA^37^ (the same V-ATPase inhibitor as BafA1 but with a different binding site) further attenuated the green fluorescent signals and significantly reduced the red fluorescence of LysoBrite (Figure 4c, Panel IV, and Figure 4d), suggesting that ConA inhibits both autolysosome formation and lysosome function.

### Repeated inhibition of eMI increases cancer cell dependence on compensatory MA

**Figure 5.**
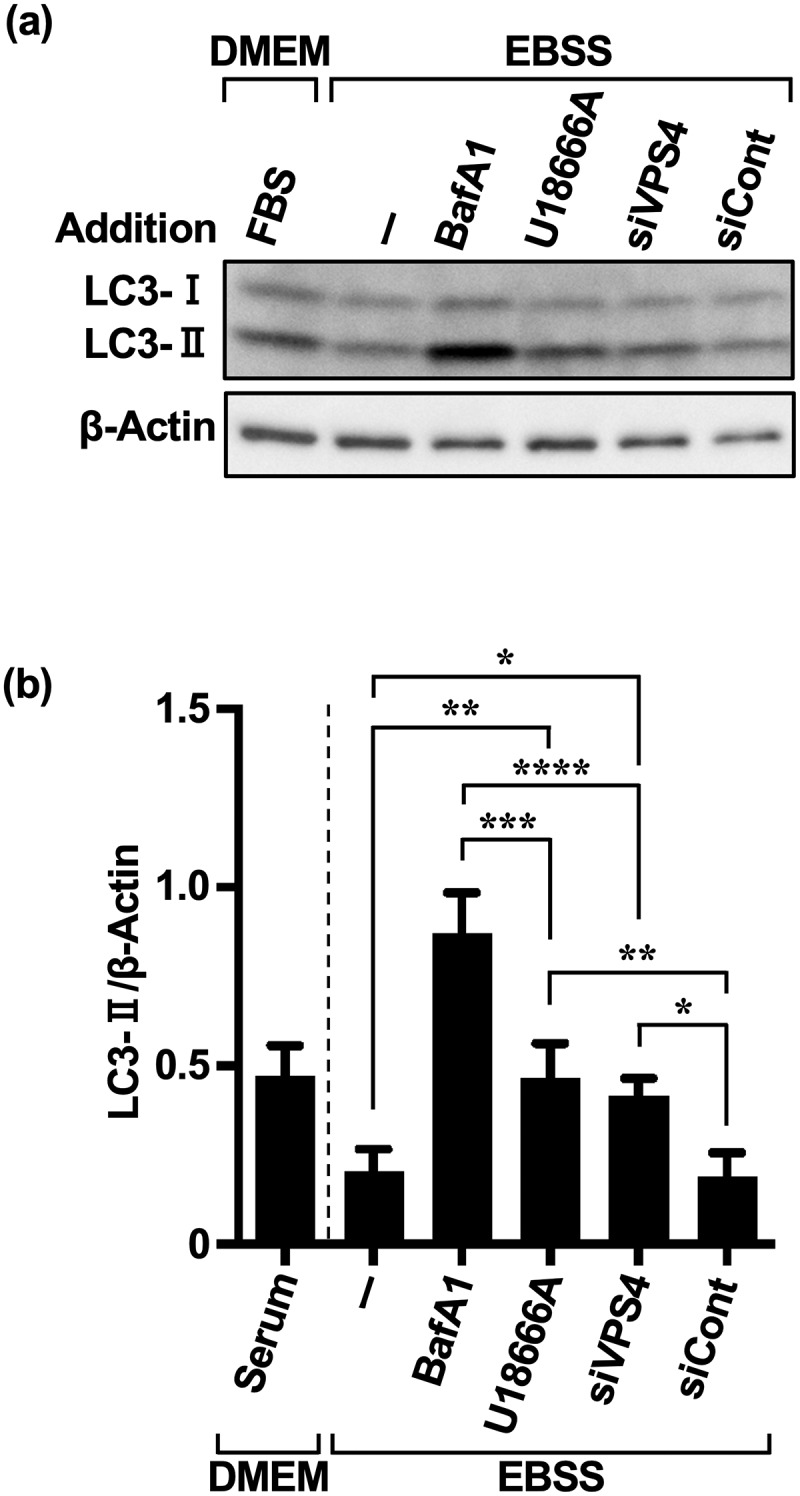
Repeated treatment with U18666A and AD in HeLa and MCF-7 cells promotes MA dependence. (a) Western blot images of HeLa cells treated three times with U18666A and AD and a fourth treatment with each inhibitor [BafA1 (50 nM), U18666A (5 μg/mL), siVPS4 (50 nM), and siCont (50 nM)] and AD. Cells were pretreated with BafA1 and U18666A for 3 and 1 h, respectively, washed, and exposed to EBSS for 5 h. Non-AD conditions were established using DMEM with 10% serum. (b) Quantification of LC3-II degradation and accumulation. Results are expressed as the mean ± SD of four independent experiments. *p < .05, **p < .01, ***p < .001 (two-way ANOVA with Tukey’s multiple comparisons test). AD, amino acid deprivation; BafA1, bafilomycin A1; EBSS, Earle’s balanced salt solution.

To examine the activation of compensatory MA by repeated treatment with U18666A and extracellular AD, cells treated with three cycles of U18666A and AD were treated with siVPS4 in conjunction with AD or BafA1 in conjunction with AD in the fourth cycle. Given that silencing by siVPS4 induces the accumulation of LC3-II derived from the eMI pathway, the detection of significantly greater LC3-II accumulation than in the base control siRNA treatment suggests that eMI may still be activated, albeit at lower levels. Furthermore, the LC3-II content following silencing with siVPS4 was 48% of total LC3-II accumulated with BafA1 treatment. Therefore, up to 52% of LC3-II accumulation could be attributed to the compensatory MA pathway (Figure 5).

We observed no significant difference in LC3-II accumulation between cells treated with U18666A and AD for three cycles and then treated with U18666A and AD or siVPS4 and AD for a fourth cycle (single:repeated = 0.46 ± 0.10:0.42 ± 0.05; Figure 5). Figure 4c then suggests that the degree of LC3-II accumulation following U18666A treatment does not directly reflect the degree of eMI pathway inhibition, but instead that U18666A treatment in HeLa cells likely inhibits autolysosome formation in the eMI pathway.

Importantly, LC3-II accumulation in cells treated with U18666A and AD once was ~ 66% of the total LC3-II accumulated (Figure 3) but was significantly reduced to ~ 48% in cells treated three times with U18666A and AD and a fourth time with siVPS4 and AD (Figure 5; Supplementary Figure S4). LC3-II accumulation in cells treated once with U18666A and AD was ~ 75% of the total LC3-II accumulated following a single treatment with BafA1 and AD (Figure 3). Moreover, LC3-II accumulation decreased to ~ 53% of the total after four cycles of repeated U18666A and AD treatments (Figure 5). The total flux of eMI and MA was defined as the overall LC3-II accumulation (100%), achieved by subjecting cells to three cycles of U18666A and AD treatment, followed by BafA1 and AD treatment in the fourth cycle.

In HeLa cells treated with three cycles of U18666A and AD followed by siVPS4 and AD or U18666A and AD in the fourth cycle, the amount of LC3-II presumably accumulated via the MA pathway increased from 34% to 52% or 25% to 47%. These results suggest that repeated inhibition of eMI in HeLa cells may increase reliance on the compensatory MA pathway.

As VPS4 depletion may also affect MA flux by preventing autophagosome – lysosome fusion,^21,30^ VPS4 depletion may affect LC3-II accumulation via the MA pathway. Based on previous results, we hypothesized that increased compensatory MA activity due to repeated treatment with U18666A and AD may promote treatment resistance in cancer cells. Thus, we investigated the relationship between compensatory MA and cell activity.

### Simultaneous inhibition of eMI and MA under intracellular starvation reduces resistance to cancer cell death via repeated eMI inhibition

To test the above hypothesis, cytotoxicity was measured based on lactate dehydrogenase (LDH) leakage from HeLa and MCF-7 cells after single or repeated treatment with U18666A and AD.

**Figure 6.**
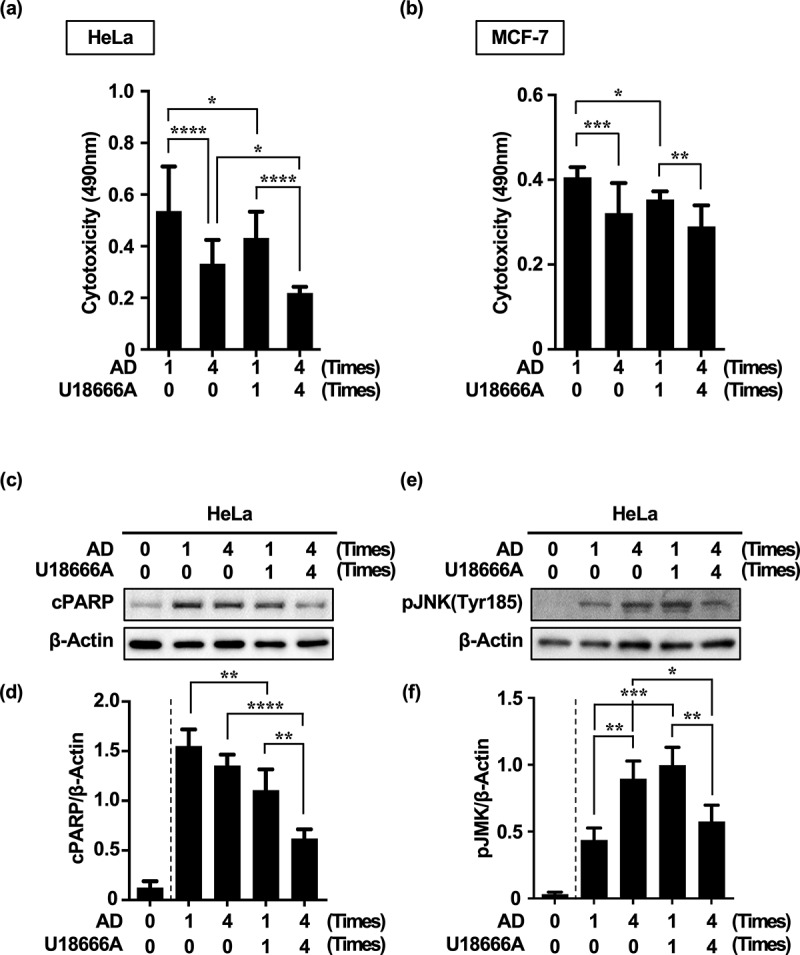
Repeated treatment with U18666A and AD in HeLa and MCF-7 cells increases resistance to cell death. (a, b) Cytotoxicity in HeLa cells (a) and MCF-7 cells (b) treated four times with U18666A and AD based on LDH activity. (c, e) Western blot images of cPARP (c) and p-JNK (Tyr185) expression (e) in HeLa cells treated four times with U18666A and AD. (d, f) Quantification of cPARP (d) and p-JNK(Tyr185) (f) expression in HeLa cells. Numbers in the graph footnotes indicate the number of U18666A or AD treatments. Results are expressed as the mean ± SD of four independent experiments. *p < .05, **p < .01, ***p < .001, ****p < .0001 (two-way ANOVA with Tukey’s multiple comparisons test). AD, amino acid deprivation; cPARP; cleaved PARP; LDH; lactate dehydrogenase; p-JNK; phosphorylated JNK.

Repeated treatment with AD alone reduced cytotoxicity more than a single dose did. Remarkably, repeated treatment with U18666A and AD significantly reduced cytotoxicity in both Hela and MCF-7 cells (Figure 6a,b). In other words, the decrease in cytotoxicity due to repeated cellular stress was consistent with an increase in MA activity (Figure 3 and 5; Figure 6a,b; Supplementary Figure S4). Therefore, we investigated the relationship between the compensatory increase in MA activity (Figure 3, 4, and 5; Supplementary Figure S4) and molecular mechanisms associated with resistance to cell death.

Based on the results thus far, eMI is also an autophagy induced by acute starvation stress, whereas compensatory autophagy is induced by chronic starvation stress. We investigated how apoptotic signaling is reprogrammed during the transition from the acute phase of starvation-induced homeostasis disruption to the chronic phase of homeostasis restoration. As indicators of reprogramming, we evaluated changes in the expression of fragmented PARP (cPARP),^38^ an indicator of the execution phase of cell death, and phosphorylated JNK (p-JNK, Tyr185),^39^ an indicator of stress response pathway activation and determinant of cell fate.

Interestingly, the expression of cPARP in AD alone hardly changed after repeated treatments (AD × 1: AD × 1 + U18 × 1 = 1.55 ± 0.17: 1.11 ± 0.21). However, even one cycle of AD treatment significantly decreased cPARP expression when combined with U18666A treatment. In particular, cPARP expression was significantly reduced in cells repeatedly treated with U18666A and AD [AD × 1 + U18 × 1: AD × 4 + U18 × 4 = 1.11 ± 0.21: 0.62 ± 0.09] (Figure 6c,d).

JNK phosphorylation levels were significantly increased under repeated treatments of AD alone as well as in combination with a single U18666A treatment. Surprisingly, repeated U18666A and AD treatments significantly decreased JNK phosphorylation levels [AD × 1 + U18 × 1: AD × 4 + U18 × 4 = 0.90 ± 0.08: 0.66 ± 0.11] (Figure 6e,f).

These findings suggest that cytotoxicity and cPARP expression were largely associated with stress intensity. However, JNK phosphorylation showed levels consistent with stress intensity in the absence of U18666A but were inversely correlated with stress intensity in the presence of U18666A. Therefore, cytotoxicity/cPARP and JNK phosphorylation levels were not necessarily correlated in the presence of U18666A.

### Simultaneous inhibition of eMI and MA under intracellular starvation reduces resistance to cancer cell death by repeated eMI inhibition

To determine whether the inhibition of compensatory MA leads to cell death in HeLa cells repeatedly treated with U18666A and AD, we measured the cytotoxicity of cells treated three times consecutively with U18666A and AD, followed by treatment with U18666A, Wtn (10 nM), and AD for the fourth cycle.

**Figure 7.**
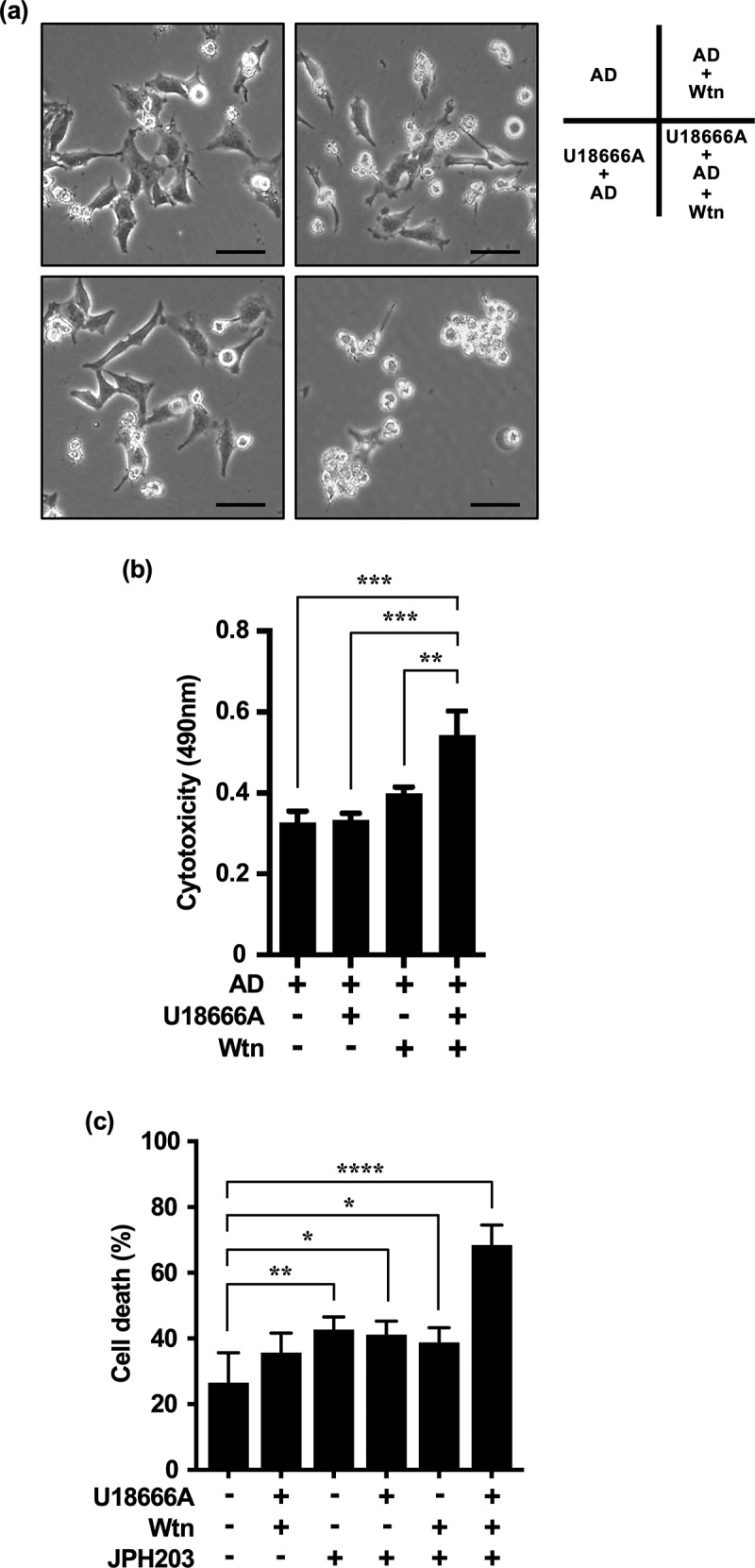
Combined effect of U18666A and Wtn on cells repeatedly treated with U18666A and AD is enhanced by LAT1 inhibition. (a, b) Cell morphology (a) and cytotoxicity assessment (b) after fourth stress exposure and three replicates U18666A and AD treatments in HeLa cells; for the fourth treatment, cells were pretreated with U18666A (2 μg/mL) for 3 h, washed, and then exposed to EBSS supplemented with Wtn (10 nM) for 8 h. For the third treatment, cells were treated with U18666A for 3 h, washed, and then exposed to EBSS supplemented with Wtn (10 nM) for 8 h. For the second treatment, cells were treated with U18666A for 3 h, washed, and then exposed to EBSS supplemented with Wtn (10 nM) for 8 h. (c) Cell death rate induced in HeLa cells after three repeated treatments with U18666A and AD and a fourth treatment with serum-free medium only; U18666A (2 μg/mL), Wtn (10 nM), and JPH203 (3 mM) alone; U18666A+JPH203, Wtn+JPH203, and U18666A+JPH203, respectively; or 12 h of incubation with Wtn+JPH203. The percentage of dead cells was quantified using trypan blue staining (*n* > 57,600 cells/condition). Results are expressed as the mean ± SD of four independent experiments. *p < .05, **p < .01, ***p < .0001 (two-way ANOVA with Tukey’s multiple comparisons test). AD, amino acid deprivation; Wtn, wortmannin.

Under non-AD (serum-free medium), Wtn showed little cell killing effect (data not shown); under AD, Wtn alone did not have a significant cell killing effect compared to the cytotoxicity of AD alone. However, Wtn and U18666A synergistically enhanced cytotoxicity when used in combination under AD (Figure 7a,b). Surprisingly, this means that inhibition of both compensatory MA and eMI, which probably act through different mechanisms, has to be performed under AD-induced cellular stress to inhibit compensatory MA induced in eMI inhibitor-resistant cancer cells.

We also hypothesized that intracellular AD promotes the effect of autophagy inhibitors because cells repeatedly and continuously treated with autophagy inhibitors under extracellular AD would have passed the immediate response phase and suffer from chronic AD. To verify the combined effects of U18666A and Wtn in intracellular AD, we focused on the inhibition of mTORC1,^40^ a nutrient sensor involved in autophagy regulation. JPH203,^40,41^ a leucine transporter, major activator of mTORC1, and the only LAT1 inhibitor currently considered in clinical trials, was used to indirectly inhibit mTORC1 function and induce intracellular AD. We attempted to induce cancer cell death using either U18666A alone, Wtn alone, or combination treatments. Cells treated with U18666A and AD for three cycles were treated for the fourth cycle with serum-free medium containing glucose and amino acids in the presence of JPH203 (3 mM, Supplementary Figure S3). Similar to the effect of the combined treatment under extracellular AD (Figure 7a), JPH203 promoted the inhibitory effect of U18666A and Wtn. However, in the absence of JPH203, the inhibitory effect of U18666A alone, Wtn alone, or in combination was limited (Figure 7c).

The control group showed a cell death rate of ~ 26%. This was probably because cells that had already undergone three cycles of U18666A and AD treatment were used as the baseline cells. In other words, repeated and continuous treatment with U18666A may have normalized cholesterol accumulation in endosomes and lysosomes and already induced chronic stress.^6,21^ Additionally, cells adapted to intracellular cholesterol accumulation and starvation by repeated and continuous treatment with U18666A and AD may have undergone excessive adaptive changes and cell death in an amino acid-rich nutrient environment.^42^

These results suggest that the combination of eMI and MA inhibitors in the presence of LAT1 inhibitors, which induce intracellular AD, represents a promising strategy for overcoming therapeutic resistance in eMI inhibitor-resistant cancer cells undergoing chronic AD. Notably, mTORC1 regulation may be essential for successful autophagy therapy in cancer cells undergoing repeated and continuous cellular stress.

## Discussion

We showed that HeLa and MCF-7 cells preferentially induced immediate and selective eMI in response to extracellular AD. We highlighted the novel possibility of an autophagic response in which cancer cells increase their resistance to cell death by complementing the dysfunction of a predominant and selective autophagy pathway with the initiation of another, presumably nonselective, autophagy pathway. While selective autophagy has the advantage of degrading specific dysfunctional cellular components and minimizing the initial damage under exposure to severe environments, including chronic starvation, cells have adaptive strategies to switch to nonselective autophagy, which modifies and removes large amounts of damaged cytoplasmic components to promote survival.^43,44^ The complementary relationship between different autophagy pathways is one explanation for the lack of long-term efficacy associated with continuous autophagy inhibitor monotherapy. Targeting of this complementary relationship may be a promising new approach. Accordingly, we targeted only late-induced compensatory MA in cells under repeated and continuous autophagy inhibition, perhaps chronically starved cells,^45^ but found that targeting only newly and compensatory-induced autophagy resulted in little cell death. Paradoxically, when intracellular AD was induced by LAT1 inhibitors, the combined use of MA and eMI inhibitors resulted in a potent cell death effect. These results suggest that combination treatment with the LAT1 inhibitor JPH203 is a promising approach for enhancing the anticancer efficacy of autophagy inhibitors. Specifically, mTORC1 inactivation, which induces intracellular starvation and nonselective MA,^45^ is essential for restoring sensitivity to autophagy inhibitors.

Repeated and prolonged metabolic or oxidative stress induced by AD and anticancer drugs selects subpopulations of dormant cancer^17^ and stem cells^18^ induced by reprogramming of these cells.^16^ A notable feature of these cancer stem-like cells (CSCs) is their maintenance of low reactive oxygen species (ROS) levels, which strongly determines their development and survival.^46^ The importance of a low ROS state in CSCs has been demonstrated in various reports. For example, CSC subpopulations of T-cell acute lymphoblastic leukemia cells and hepatocytes were eliminated by aldehyde dehydrogenase and glutathione peroxidase inhibitors,^47,48^ and the sphere-forming ability of hepatic CSCs was suppressed by the paraquat-induced upregulation of ROS levels.^49^ It is possible that cells chronically starved through repeated and continuous treatment with U18666A and AD have established resistance and been reprogrammed with CSC-like characteristics. Accordingly, CSC-like cells may use compensatory autophagy as a defense strategy to support their superior viability.^8^ It makes sense then that CSC-like cells rely on amino acid transporters such as glutamine/glutamate transporters and LAT1 to adapt to chronic starvation/oxidative stress and maintain redox and energy homeostasis. These amino acid transporters are closely involved in glutamine/glutamate metabolism and mTORC1 signaling, which are key mechanisms of reduced glutathione and ATP production.^50^ Therefore, the indirect targeting of mTORC1, which is closely associated with the nonselective autophagy pathway, with amino acid transporter and autophagy inhibitors represents a promising treatment option for refractory cancers, including resistant dormant cells.^51^

Recently, activation of eMI-,^52^ chaperone-,^1^ and micropinocytosis-mediated^53^ compensatory autophagy on behalf of MA has been reported in *Drosophila* fibroblasts, mouse fibroblasts, and human pancreatic ductal adenocarcinoma cells, respectively. Compensatory autophagy mechanisms that complement the reduced function of different autophagy pathways, including those reported in this study, may be key in cancer cell homeostasis. Incidentally, compensatory MA induced by repeated and continuous inhibition of eMI was associated with changes in JNK activity in response to stress intensity. We observed a complex relationship between the level of cytotoxicity and apoptotic activity (cPARP) induced by repeated treatment with AD alone or with U18666A and AD, and JNK activity (Figure 6). This may be due to the dual regulatory mechanism of JNK in cell fate, either apoptosis or autophagy, depending on its situational phosphorylation status, and overlying environmental conditions, such as oxidative stress induced by U18666A.^54^

Cytotoxicity was significantly reduced when U18666A treatment was combined with the single or single/repeat treatments of AD (Figure 6a,b). Notably, the reduction in cytotoxicity with combined U18666A treatment was contextually consistent with the reduction in cPARP levels (Figure 6c). This is consistent with a previous report showing that mitochondrial membrane stabilization and inhibition of Bax activation by U18666A treatment promoted apoptosis resistance in HeLa cells.^28,55^ This may reflect an adaptive response to increased stress intensity: the synergistic induction of compensatory MA (Figure 2 and 3) and inhibition of apoptosis^55^ brought about by U18666A. Inverse to the cytotoxicity and cPARP profiles, p-JNK levels increased with increasing cellular stress intensity. However, this was reversed when the stress intensity was increased by repeated treatment with U18666A and AD, with a concomitant decrease in cytotoxicity and cPARP levels (Figure 6f). This complex event may stem from an interaction between the two-sided biogenic effects of JNK, which promotes pathological cell death or inappropriate cell survival, and the two-sided effects of U18666A on cell viability. U18666A is known to accumulate in the lysosomes and mitochondrial membranes of many tumor cells, particularly in cultured tumor cells, promoting membrane stability as well as the production of ROS.^56,57^ In other words, JNK may either be hyperphosphorylated to transiently activate the JNK signaling pathway toward cell death signals^54,56^ or the inhibition of apoptosis (another effect of U18666A) and enhancement of the stress adaptation response (through activation of the Beclin-1 dissociation pathway from the Bcl-2/Beclin-1 complex by JNK hypoactivation)^4,58^ may induce compensatory MA and promote cell survival.^59^ In addition, the ability of tumor cells to increase the membrane stability of mitochondria and lysosomes involved in cell fate through the cholesterol-accumulating effects of U18666A may be a biological defense against factors that inhibit proton leakage^57^ and cause membrane disruption.^56^ Notably, excessive membrane stiffness is not necessarily advantageous from an energy metabolism standpoint, as it can lead to reduced membrane fluidity, potential,^60^ and lysosomal enzyme activity.^61^

In conclusion, cancer cells may induce compensatory MA as an adaptive response to chronic starvation under repeated and continuous inhibition of constitutive eMI, which presumably contributes to cellular homeostasis. This adaptive response appears to be an important survival mechanism under nutrient-limited conditions and oxidative stress, especially given that both eMI and compensatory MA were effectively inhibited by JPH203. Therefore, the adaptive response is closely related to intracellular starvation and the function of the nutrient sensor mTORC1. The induction of compensatory MA by repeated U18666A- and AD-induced stress may promote or impede survival based on the dual effects of U18666A and JNK on cell viability and death. This study provides a useful reference for the development of new therapeutic strategies that limit adaptive responses to autophagy inhibition by effectively blocking the nutrient-acquisition pathways of cancer cells. We showed that eMI, a selective autophagy induced by extracellular AD, can switch to nonselective bulk MA. However, the relationship between intracellular cholesterol concentration and JNK phosphorylation and the effects of JNK and various amino acid transporter inhibitors on the regulation of this switch are unclear and require further investigation.

## Materials and methods

### Reagents

Unless otherwise noted, all reagents were purchased from Fujifilm Wako Pure Chemical Corporation. Immunostar LD (296–69901) and DMEM with glucose (041–29775) were purchased from Fujifilm Wako Pure Chemical Corporation. EBSS (E7510) and DMEM without glucose (A1443001) were purchased from Sigma-Aldrich. U18666A (10009085) and ConA (11050) were purchased from Cayman Chemical. We purchased 2-deoxy-D-glucose (2-DG) from Nacalai Tesque. HCQ (FH24322) was purchased from Biosynth. BafA1 (S1413) and Wtn (KY12420) were purchased from Selleck Biotech Corporation. JPH203-sulfobutylether-β-cyclodextrin (SBECD; JPF1601) was kindly provided by J-Pharma; JPH203 was mixed with SBECD at a mass ratio of 1:2.4 to make it water-soluble.

### Cells

Human cervical cancer HeLa cells were purchased from the American Type Culture Collection. Cells of the human mammary carcinoma-derived cell line MCF-7 were purchased from the Cell Bank of the Medical Cell Resource Center for Biomedical Research, Institute of Development, Aging and Cancer, Tohoku University.

### Antibodies

Regarding primary antibodies, VPS4A/B polyclonal antibody (1:1,000; 17673–1-AP) and Phospho-JNK (Tyr185) mAb (1:3,000; 80024–1-RR) were purchased from Proteintech. Cleaved PARP (Asp214) polyclonal antibody (pAb; 1:1,500; #9541) was purchased from Cell Signaling Technology. We purchased p62 (SQSTM1) mAb (1:1,500; M162–3) and anti-LC3 mAb (1:1,500; M186–3) from Medical & Biological Laboratories. Anti-β-actin mAb (1:10,000; A2228) and anti-beta-tubulin pAb (1:6,000; T2200) were purchased from Sigma-Aldrich and used as loading control. The secondary antibodies HRP-conjugated anti-mouse-IgG Ab (1:12,000; 115-035-166) and HRP-conjugated goat anti-rabbit IgG (1:12,000; 111-035-144) were purchased from Jackson ImmunoResearch Laboratories.

### Cell culture and drug stimulation

HeLa and MCF-7 cells were cultured in DMEM (4.5 g/L nutrient medium) containing glucose supplemented with 10% fetal bovine serum and 100 U/mL penicillin – streptomycin at 37°C in a 5% CO_2_ environment. To inhibit lysosomal function, cells were incubated in EBSS supplemented with HCQ (100 μM), Wtn (10 nM), ConA (5 nM), and BafA1 (50 nM), respectively. For U18666A treatment, HeLa and MCF-7 cells were pretreated with 5 and 8 μg/mL U18666A for 3 h, respectively, followed by washing and AD treatment.

### Amino acid and glucose deprivation

For each experiment, cells were cultured for 36 h to 60% confluency; AD was performed by exposure to EBSS.

### Establishment of repeated AD-experienced cells as well as eMI-inhibited and repeated AD-experienced cells

To establish AD-experienced cells, they were exposed to EBSS for 5 h and cultured in DMEM for 3 d to recover. The cells were passaged and exposed again for a total of three times and used for validation. To establish experienced cells for repeated eMI inhibition and AD, HeLa and MCF-7 cells were pretreated with 5 and 8 μg/mL U18666A, respectively, for 3 h, followed by washing and exposure to AD for 5 h, and a final 3-d culture in a nutrient medium for recovery. Thereafter, cells were passaged and pretreated with U18666A for 3 h twice (2 and 8 μg/mL U18666A were used for HeLa and MCF-7 cells, respectively). Cells subjected to three repetitions of AD with eMI inhibition were used for validation.

### Western blot analysis

Total protein was extracted using 1 mM MgCl_2_, 10 mM HEPES-OH (pH 7.55), 0.25 M NaCl, 5% glycerol, 0.15% Tx-100, 20 μg/mL DNaseI, and a protease inhibitor cocktail. Cell extracts (3 μg/well) were denatured with SDS buffer, separated using SDS-PAGE, and transferred to an Immun-Blot PVDF membrane (162–0177) using a wet transfer system (80 V, 50 min). After blocking with 3% skim milk, primary and secondary antibodies were reacted at 25°C for 60 min. Proteins were detected using the luminescent reagent Immunostar LD, and the emitted signals were imaged using a high-sensitivity chemiluminescence imaging system (WSE-6100 H-CS LuminoGraph, Ato Corporation). Owing to the inevitably high levels of autophagy marker proteins in cancer cells at 0 h under AD (immediately after changing the nutrient medium to EBSS) and non-AD (nutrient medium) conditions, we present the changes in their expression as a percentage of the control-group expression.

### siRNA gene silencing

VPS4A/B siRNA (siVPS4A/B) and negative control siRNA were purchased from Nippon Gene. The siVPS4 sequence (984–1002 bp, 5′-AUUUGCACUAGGAACUCCG-3′) on the VPS4B sequence (NM_004869) was selected to target and silence VPS4B. This sequence has a mismatched sequence site (874–892 bp) at 4/19 bases in the VPS4A sequence (NM_013245), which is in a paralogous relationship (Supplementary Figure S2a). Lipofectamine 2000 was used for siVPS4 transfection 48 h before AD or inhibitor treatment, according to the manual (Thermo Fisher Scientific). As the silencing efficiency of siVPS4 before inhibitor treatment was ~ 100% at 50 nM (Supplementary Figure S2b) and little cytotoxicity was observed (data not shown), we selected 50 nM siVPS4 for the experiments.

### Quantification of autolysosomes and lysosome fusion

DALGreen (Ex/Em = 450–490/520 nm) and LysoBrite (Ex/Em = 450–490/520 nm) labeling reagents were used to detect autolysosomes and lysosomes, respectively. Samples were imaged using a fluorescence microscope (Eclipse TE2000-U, Nikon) equipped with a Peltier two-stage cooled CMOS camera (FL-20, BioTools). Two researchers were tasked to detect dot structures in a blinded manner. The results are expressed as the percentage of the total number of cells used to quantify the number of autolysosome-expressing cells. Cells were identified using 4′,6-diamidino-2-phenylindole (DAPI) staining.

### Cytotoxicity evaluation

The cell culture medium was collected and centrifuged (12,000 rpm, 20 min, 4 °C). The amount of LDH in the supernatant was determined using a Cytotoxicity LDH Assay Kit-WST (341–91754, Dojindo Laboratories).

### Statistical analysis

Experiments were conducted independently at least three times. Unless otherwise noted, multiple comparisons were analyzed using one-way or two-way analysis of variance, followed by Dunnett’s or Tukey’s multiple comparison tests. Binomial comparisons were analyzed using the Wilcoxon rank-sum test in Prism v.7.0d software (GraphPad Software). Data are expressed as the mean ± SD for all analyses, and *p* < .05 was considered statistically significant.

## Ethics approval

All experimental protocols were reviewed and approved by the Animal and Human Experimentation Ethics Committee of Tokushima University (No. 2021–77).

## Supplementary Material

Text_for_supplemental_materials.docx

Supplementary Figure 1.tiff

Supplementary_Figure_4.tiff

Supplementary Figure 3.tiff

Supplementary Figure 2.tiff

## Data Availability

The authors hereby confirm that the data supporting the conclusions of this study are included in the manuscript and its supplementary materials.
