# Supplementary material for: Amino acid deprivation in cancer cells with compensatory autophagy induction increases sensitivity to autophagy inhibitors
Source: Mol Cell Oncol. 2024 Jul 14;11(1):2377404. doi: 10.1080/23723556.2024.2377404 (PMC11253891; doi:10.1080/23723556.2024.2377404)
Supplement: Text_for_supplemental_materials.docx [file KMCO_A_2377404_SM9694.docx]

**Supplementary material figure legends**

**Supplementary Figure 1: Acute phase autophagy by extracellular AD in HeLa and MCF-7 cells involves an autophagy pathway distinct from MA**

Western blot images of LC3-II and p62 changes over time in response to extracellular AD in HeLa and MCF-7 cells to assess changes in degradation and accumulation of LC3-II (a, e) and p62 (c, g) over time after AD in the absence and presence of ConA (10 nM), respectively. Western blot images of HeLa and MCF-7 cells were analyzed using 3 μg of cell extract per well. (b, d, f, h) Quantification of LC3-II (b, f) and p62 (b, h) degradation (dashed line) and accumulation (solid line) over time. Results are expressed as mean ± SD of three independent experiments. Significance was calculated with two-way ANOVA with Sidak’s multiple comparisons test (***p < 0.001, ****p<0.0001)**.** AD, amino acid deprivation; HCQ, hydroxychloroquine.

**Supplementary Figure 2: siRNA sequences for VPS4 gene silencing and their suppressive effects.**

(a) For the siRNA (siVPS4) sequence, a sequence (5'-auuugcacuaggaacuccg-3') located between 984 and 1002 bp on the VPS4B sequence (NM_004869) was selected to target and silence VPS4B. This sequence was a mismatched site at a ratio of 4/19 bases to the sequence present between 874 and 892 bp on VPS4A (NM_013245), which is in a paralogous relationship. Since the silencing efficiency of siVPS4 was nearly 100% at 50 nM (b) and no cytotoxicity was observed, 50 nM siVPS was used in the experiments. Results are expressed as mean ± SD of three independent experiments. Significance was calculated with one-way ANOVA with Tukey’s multiple comparisons test (*p < 0.05, **p < 0.01).

**Supplementary Figure 3: Cytotoxicity curve of JPH203**

For cytotoxicity, HeLa cells (80% cell density) treated in triplicate with U18666A and AD were treated with SFM (a) or SBECD (b) with JPH203 at the concentrations shown in the figure for 12 h, and the cell culture medium was collected. After centrifugation (12,000 rpm, 20 min, 4 °C), the amount of lactate dehydrogenase (LDH) in the supernatant of the culture medium was used for quantification. For experiments, 3 mM JPH203 was mixed with SBECD at a mass ratio of 1:2.4 to make it water-soluble. Results are expressed as mean ± SD of four independent experiments. Significance was calculated with one-way ANOVA with Dunnett’s multiple comparisons test (*p < 0.05, **p < 0.01, ****p < 0.0001). SFM, serum free medium; SBECD, sulfobutyl ether beta-cyclodextrin.

**Supplementary Figure 4: Repeated treatment with U18666A and AD increases reliance on the MA pathway to compensate for reduced eMI activity.**

When the eMI pathway was selectively inhibited using siVPS4, eMI accounted for 66% of all autophagy in HeLa cells under AD. However, after three rounds of U18666A and AD treatment, eMI dependence dropped to 48% when siVPS4 + AD was used as the fourth treatment (Figure 3). However, MA increased by 153%, from 34% to 52% (Figure 5). This suggests that repeated treatment with U18666A and AD may activate the MA pathway to compensate for the reduced eMI pathway.
